# The Teleost Thymus in Health and Disease: New Insights from Transcriptomic and Histopathological Analyses of Turbot, *Scophthalmus maximus*

**DOI:** 10.3390/biology9080221

**Published:** 2020-08-13

**Authors:** Paolo Ronza, Diego Robledo, Ana Paula Losada, Roberto Bermúdez, Belén G. Pardo, Paulino Martínez, María Isabel Quiroga

**Affiliations:** 1 Departamento de Anatomía, Producción Animal y Ciencias Clínicas Veterinarias, Universidade de Santiago de Compostela, 27002 Lugo, Spain; anapaula.losada@usc.es (A.P.L.); roberto.bermudez@usc.es (R.B.); misabel.quiroga@usc.es (M.I.Q.); 2 The Roslin Institute and Royal (Dick) School of Veterinary Studies, University of Edinburgh, Midlothian EH25 9RG, UK; diego.robledo@roslin.ed.ac.uk; 3 Instituto de Acuicultura, Universidade de Santiago de Compostela, 15782 Santiago de Compostela, Spain; belen.gomez@usc.es (B.G.P.); paulino.martinez@usc.es (P.M.); 4 Departamento de Zoología, Genética y Antropología Física, Universidade de Santiago de Compostela, 27002 Lugo, Spain

**Keywords:** fish, T lymphocytes, infection, malnutrition, inflammation, aquaculture, RNA-Seq, histopathology, immunohistochemistry, enteromyxosis

## Abstract

The thymus is a primary lymphoid organ that plays a pivotal role in the adaptive immune system. The immunobiology of the thymus in fish is considered to be similar to that of mammals, but it is actually poorly characterized in several cultured teleost species. In particular, while investigations in human and veterinary medicine have highlighted that the thymus can be affected by different pathological conditions, little is known about its response during disease in fish. To better understand the role of the thymus under physiological and pathological conditions, we conducted a study in turbot (*Scophthalmus maximus*), a commercially valuable flatfish species, combining transcriptomic and histopathological analyses. The myxozoan parasite *Enteromyxum scophthalmi*, which represents a major challenge to turbot production, was used as a model of infection. The thymus tissues of healthy fish showed overrepresented functions related to its immunological role in T-cell development and maturation. Large differences were observed between the transcriptomes of control and severely infected fish. Evidence of inflammatory response, apoptosis modulation, and declined thymic function associated with loss of cellularity was revealed by both genomic and morphopathological analyses. This study presents the first description of the turbot thymus transcriptome and provides novel insights into the role of this organ in teleosts’ immune responses.

## 1. Introduction

The thymus is a primary lymphoid organ that plays a critical and unique role in the development of T cells. This organ provides the necessary microenvironment for the precursors of lymphocytes to proliferate, rearrange their receptors, and finally differentiate into mature T lymphocytes [1,2,3]. Once these cells have gone through the processes of selection and differentiation, they leave the thymus and migrate to the periphery to perform their major role in the body’s immune response [4,5].

The immunobiology of this organ, particularly its pivotal role in the adaptive immune response as the main site of T-cell development, is considered to be similar in fish and mammals [6,7]. Despite its relevance as a primary lymphoid organ and its potential importance in the response to different diseases affecting aquaculture production, the characterization of the thymus in several cultured teleost species has scarcely been addressed to date. In particular, little is known about its behavior in pathological conditions. This is not surprising, since it is still considered an underexplored organ even in human medicine, mainly due to the difficulty of accessing in [8]. Similarly, in fish the thymus is usually not included in routine tissue sampling, probably also because of the difficulties due to its appearance and localization.

In terrestrial species, it has been demonstrated that different pathological conditions, such as malnutrition and infectious diseases, can affect the thymus, disrupting its architecture and function and having a detrimental effect on the ongoing immune response [9,10,11]. The underlying pathogenic mechanisms are beginning to be understood, mainly through studies in human and murine models [9,12,13,14].

The thymus in fish usually appears as a paired organ located in the dorsal region of each gill chamber [6,15]. In turbot, *Scophthalmus maximus*, a commercially important flatfish species, the gross anatomy and histology of the organ in juvenile fish was described by Vigliano et al. [16]. In this species, a zonation of thymus resembling the division in the cortex and medulla of mammals was reported: lymphoblasts and macrophagic cells were mainly observed in the inner part, whereas thymocytes were mostly found in the outer region [16,17]. Nevertheless, information on the function of this organ is still scarce, starting from the lack of high-throughput gene expression profiling, and its integration with morphological analysis to further understand the function of the thymus in health and disease. In this sense, the role of the thymus in response to infectious diseases has not been studied in turbot.

Enteromyxosis, caused by the myxozoan *Enteromyxum scophthalmi*, is a fast-spreading parasitic disease with a great impact on turbot aquaculture. *E. scophthalmi* is transmitted directly between animals, triggering a cachectic syndrome associated with poor growth performance and high mortality rates [18]. The main target organ is the gastrointestinal tract, where the parasite localizes into the lining epithelium, causing catarrhal enteritis of increasing severity throughout the disease. Further, the main lymphohematopoietic organs, kidney and spleen, develop severe cell depletion with the progression of the infection [19]. Recently, significant advances in our understanding of the pathogenesis of this parasitosis have been made using a multidisciplinary approach combining histopathology and RNA-seq transcriptomics [20,21]. The available data strongly indicate that turbot show a dysregulated and ineffective immune response against this parasite [18]. Nevertheless, the role of the thymus has never been evaluated in enteromyxosis.

Here, we employed a multidisciplinary approach to improve the knowledge of this primary lymphoid organ in teleosts under physiological and pathological conditions. We analyzed the transcriptomic profiles and performed histopathological studies of the thymuses of healthy and experimentally infected turbot, using enteromyxosis as the disease model.

## 2. Materials and Methods

### 2.1. Experimental Design and Histopathology

The experimental design was described in a previous paper (see Reference [20]). Briefly, 120 juvenile turbot (150 g mean weight) were divided into 55 receptor and 65 control fish and kept in two 500 L tanks per group with 5 μm filtered and UV-irradiated open-flow seawater (37.5‰ salinity) at 19 ± 1 °C at the facilities of the Instituto de Acuicultura de Torre la Sal (IATS, Cabanes, Castellón, Spain). 

The experimental infection was carried out by oral route; receptor fish received 1 mL of intestinal scraping homogenates in Hank’s balanced salt solution (HBSS) from 20 donor fish containing live *E. scophthalmi* parasites, whereas control fish were inoculated with the same amount of HBSS alone. Donor turbot came from an experimentally infected stock maintained at IATS (Castellón, Spain). Tissue samples from control and infected fish were collected at 7, 24, and 42 days postinoculation (dpi) and were preserved in Bouin’s fluid and RNAlater for histopathological and transcriptomic analyses, respectively. 

The health status of control and infected fish was assessed by light microscopy on H&E- and toluidine-blue-stained sections. Challenged fish were classified into three groups (slightly, moderately, and severely infected) according to the histopathological grading described by Bermúdez et al., which considers the lesional degree and parasite burden [19]. For the immunohistochemical study, five slightly infected turbot and five control turbot were selected at 24 dpi, and five severely infected and five control turbot at 42 dpi. Of those animals, the thymus samples from four control (two pools) and three infected fish from each time point were also RNA-sequenced. 

The experiment was carried out following international (Directive 2010/63/EU, on the protection of animals used for scientific purposes), national (Royal Decree RD1201/2005, for the protection of animals used in scientific experiments), and institutional regulations (CSIC, IATS Review Board and Institutional Animal Care and Use Committee of the University of Santiago de Compostela).

### 2.2. Immunohistochemistry 

Thin sections (3 µm thick) were placed on slides treated with silane to improve section adherence and dried overnight at 37 °C. Immunohistochemical markers of cell proliferation (proliferating cell nuclear antigen, PCNA), apoptosis (active caspase-3), and T cells (CD3ε) were used, together with antibodies against the proinflammatory molecules tumor necrosis factor alpha (TNF-α) and inducible nitric oxide synthase (iNOS) (Table 1). The anti-CD3ε antibody, generously donated by Dr. Erin Bromage (University of Massachusetts Dartmouth, USA), was raised using as an immunogen a synthetic peptide consisting of 14 amino acids from the cytoplasmic tail region of rainbow trout CD3ε [22]. The sequence alignment for the immunogenic peptide and its turbot homologue showed 85% identity (Supplementary File 1). All the other antibodies employed were commercial and were previously used in turbot tissues (see references in Table 1).

Endogenous peroxidase activity was quenched by incubation with a peroxidase-blocking solution (Dako, Glostrup, Denmark). Background prevention and antigen retrieval were performed as required. After incubation with primary antisera, slides were incubated with peroxidase (HRP)-labeled polymer conjugated to rabbit secondary antibody and peroxidase reaction was developed using a diaminobenzidine-positive chromogen (EnVision+ System-HRP kit, No. K 4011; Dako, Glostrup, Denmark). All incubations were performed in a humid chamber at room temperature. Finally, sections were counterstained with hematoxylin, dehydrated, and coverslipped with DePeX mounting medium. The sections were washed three times for 5 min in 0.1 M phosphate-buffered saline containing 0.05% Tween-20 between all subsequent steps, and positive and negative controls were included in each assay to assess its specificity. 

### 2.3. Transcriptome Analysis 

RNA extraction was performed using the RNeasy mini kit (Qiagen, Hilden, Germany) with DNase treatment following the manufacturer’s instructions. RNA quality and quantity were evaluated in a Bioanalyzer (Bonsai Technologies, Madrid, Spain) and in a NanoDrop^®^ ND-1000 spectrophotometer (NanoDrop^®^ Technologies Inc., Wilmington, DE, USA), respectively. Samples were barcoded and prepared for sequencing by the Wellcome Trust Centre for Human Genetics (Oxford, UK) on an Illumina HiSeq 4000 (Illumina Inc., San Diego, CA, USA) as 150 bp paired-end reads. 

Raw sequencing data were deposited in NCBI’s Short Read Archive (SRA) under BioProject ID PRJNA623212. The quality of the sequencing output was assessed using FastQC v.0.11.5 (http://www.bioinformatics.babraham.ac.uk/projects/fastqc/). Quality filtering and removal of residual adaptor sequences were conducted on read pairs using Trimmomatic v.0.38 [27]. Specifically, residual Illumina-specific adaptors were clipped from the reads, leading and trailing bases with a Phred score less than 20 were removed, and the reads were trimmed if a sliding window average Phred score over four bases was less than 20. Only reads where both pairs had a length greater than 36 bp after filtering were retained. 

Filtered reads were mapped to the most recent turbot genome assembly (GenBank accession No. GCA_003186165.1; [28]) using STAR v.2.7.0e [29] two-pass mode and the following parameters: the maximum number of mismatches for each read pair was set to 10% of trimmed read length, and minimum and maximum intron lengths were set as 20 bases and 1 Mb, respectively. Alignment files were assembled into potential transcripts using Cufflinks v.2.2.1 [30]. Uniquely mapped paired-end reads were counted and assigned to genes using FeatureCounts v.1.6.4 [31] and statistical analyses related to differential expression were performed using R v.3.5.2 (http://www.R-project.org/). Gene count data were used to estimate differential gene expression using the Bioconductor package DESeq2 v.3.4 [32]. Transcript abundances were calculated as transcripts per million (TPM). Gene ontology (GO) enrichment analyses were performed using Blast2GO v.4.1 [33] and Kyoto Encyclopedia of Genes and Genomes (KEGG) enrichment analyses were performed using KOBAS v3.0.3 [34]. GO and KEGG enrichments for specific gene lists were tested by comparison to the whole transcriptome of the turbot using Fisher’s exact test, and those terms or pathways showing a Benjamini–Hochberg FDR-corrected *p*-value < 0.05 were considered to be enriched.

## 3. Results

### 3.1. Histopathological Evaluation of Thymuses 

**Figure 1:**
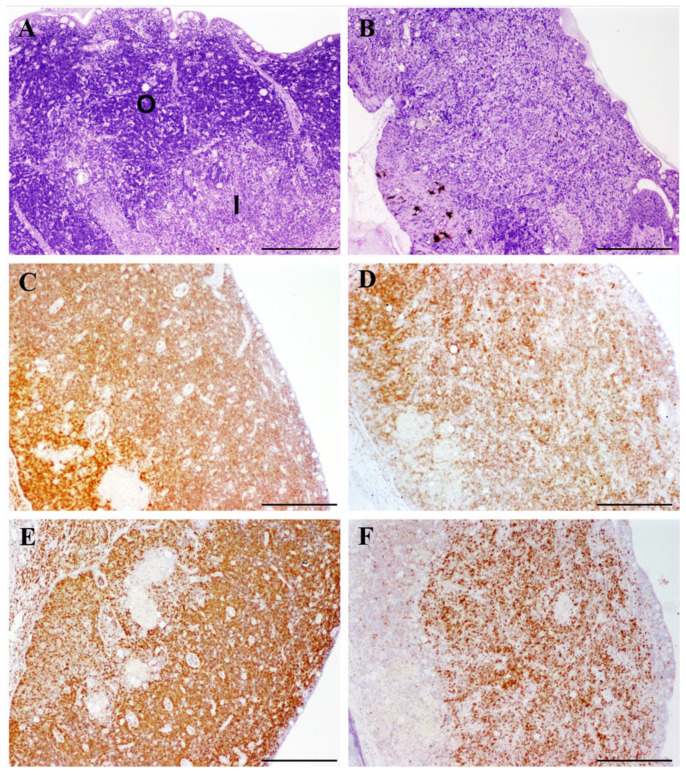
Comparative photomicrographs of thymus from healthy turbot (**A**,**C**,**E**) and turbot with severe infection by *Enteromyxum scophthalmi* (**B**,**D**,**F**). Scale bars = 200 µm. (**A**,**B**). Stained with H&E. Note that the zonation of the thymus into inner (I) and outer (O) parts was indistinguishable in the infected specimen, which also showed a reduced density of deeply basophilic thymocyte-like cells. (**C**,**D**) Immunostained for the T-cell marker CD3ε. The thymus tissues of diseased fish showed a reduced immunoreactivity, indicative of a loss of cellularity. (**E**,**F**) Immunostained for the cell proliferation marker PCNA (proliferating cell nuclear antigen). The strong immunolabel observed in the healthy fish was largely reduced during severe enteromyxosis.

The histological evaluation of the thymuses did not reveal any significant lesion in controls or in experimentally infected fish graded as slightly or moderately infected. Conversely, most fish with severe infections showed some degree of lymphocyte depletion in the outer zone of the organ, sometimes accompanied by an alteration of tissue architecture, namely a loss of differentiation between the inner and outer zones (Figure 1A,B).

### 3.2. Immunohistochemistry 

The immunohistochemistry for CD3ε and PCNA was strongly positive in healthy (control) thymus, especially in the outer zone, where most labeled cells were consistent with thymocytes (Figure 1C,E). No differences were found between slightly infected and control fish, while reduced numbers of thymocyte-like cells immunoreactive to both markers were observed in turbot with severe enteromyxosis (Figure 1D,F). 

**Figure 2:**
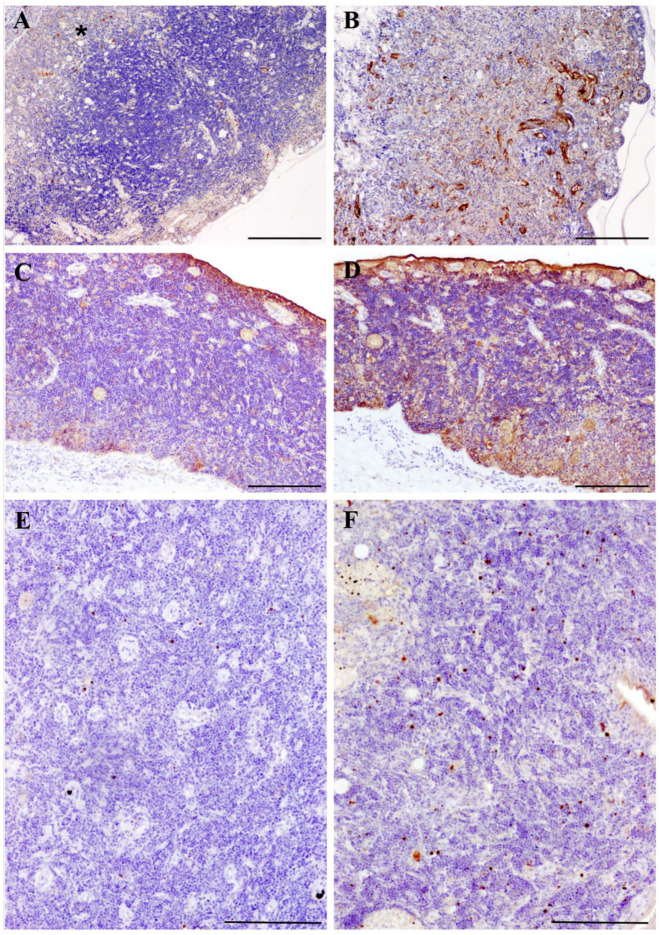
Comparative photomicrographs of thymus tissues from healthy turbot (**A**,**C**,**E**) and turbot with severe infection by *Enteromyxum scophthalmi* (**B**,**D**,**F**). (**A**,**B**) Immunostained for tumor necrosis factor alpha (TNFα), scale bars = 200 µm. Scattered macrophage-like cells positive to TNFα were present in the inner region of the thymus tissues (star) of healthy fish, while the severely infected turbot showed broader immunoreactivity to the cytokine, also found in the reticulo-epithelial network of the thymic stroma. (**C**,**D**) Immunostained for inducible nitric oxide synthase (iNOS), scale bars = 100 µm. Note that the immunostaining to this proinflammatory molecule was more diffuse in the thymus of the diseased specimen. (**E**,**F**) Immunostained for active caspase-3, scale bars = 100 µm. Higher numbers of thymocyte-like cells positive to this marker of apoptosis were noted in the outer part of the thymus of the *E.-scophthalmi*-infected fish. * shows the location of the inner region of the thymus: Scattered macrophage-like cells positive to TNFα were present in the inner region of the thymus tissues (star).

The immunochemical staining pattern of TNF-α in turbot thymus under physiological conditions has been previously described [26]. Accordingly, we observed some TNF-α-immunoreactive macrophage-like cells randomly distributed in the stroma of the organ in control (Figure 2A) and slightly infected fish. Conversely, severely infected fish showed increased immunoreactivity to the cytokine, with higher number of positive macrophage-like cells and/or enhanced staining of the reticulo-epithelial cellular network of the thymic stroma (Figure 2B).

Similarly, few iNOS-immunoreactive cells were found in the thymus tissues of control and slightly infected turbot. The immunoreactivity was mainly observed in the epithelial layer and in the cytoplasm of some mucous and macrophage-like cells (Figure 2C). Severely infected fish displayed a stronger and more diffuse immunostaining for iNOS, also observed in the thymic stroma, with a greater number of labeled macrophage-like and mucous cells (Figure 2D). 

Regarding the apoptotic marker active caspase-3, the immunolabeling was mainly detected in scattered cells consistent with thymocytes in the outer zone of the organ. The result was similar when comparing control and slightly infected turbot, while a mild increase of caspase-3 was detected in severely infected fish, which showed a higher density of positive thymocyte-like cells (Figure 2E,F). 

### 3.3. Thymus Transcriptome of Healthy Fish

**Figure 3:**
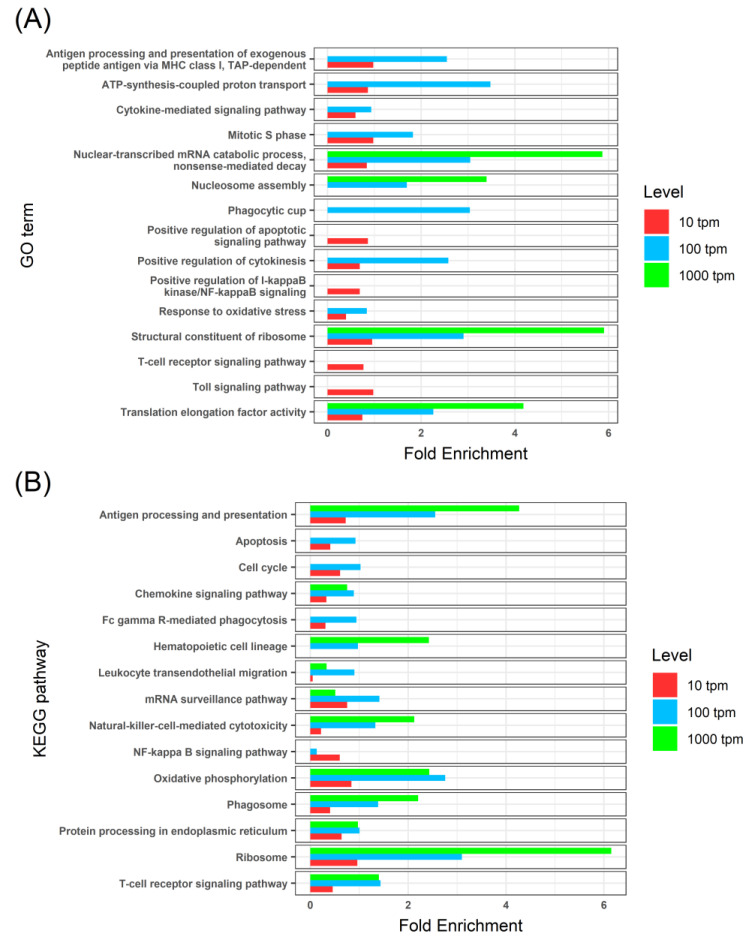
Bar graphs of representative enriched gene ontology (GO) terms (**A**) and KEGG pathways (**B**) found in three subsets of the most expressed genes in the thymus tissues of healthy turbot, tested by comparison to the whole transcriptome of the turbot. The subsets were selected based on different expression thresholds: TPM (transcripts per million) > 1000 (red, 134 genes), TPM > 100 (blue, 1322 genes) and TPM > 10 (green, 10371 genes). GO terms or pathways showing a Benjamini–Hochberg FDR-corrected *p*-value < 0.05 were considered to be enriched.

To characterize the turbot thymus transcriptome, we studied different subsets of highly expressed genes, selected based on their expression levels in control animals (TPM > 1000, 100, and 10), and performed GO term and KEGG pathway enrichment analyses (Supplementary File 2). The enrichment analyses revealed several overrepresented functions related to energy production, protein synthesis, and cell proliferation (Figure 3). Different GO terms and KEGG pathways related to immunological functions were found, such as chemokine signaling, antigen processing and presentation, T-cell receptor signaling pathways, and natural-killer-mediated cytotoxicity. Programmed cell death (i.e., apoptosis) was also overrepresented in both enrichment analyses.

### 3.4. Enteromyxosis-Induced Transcriptomic Changes

**Figure 4:**
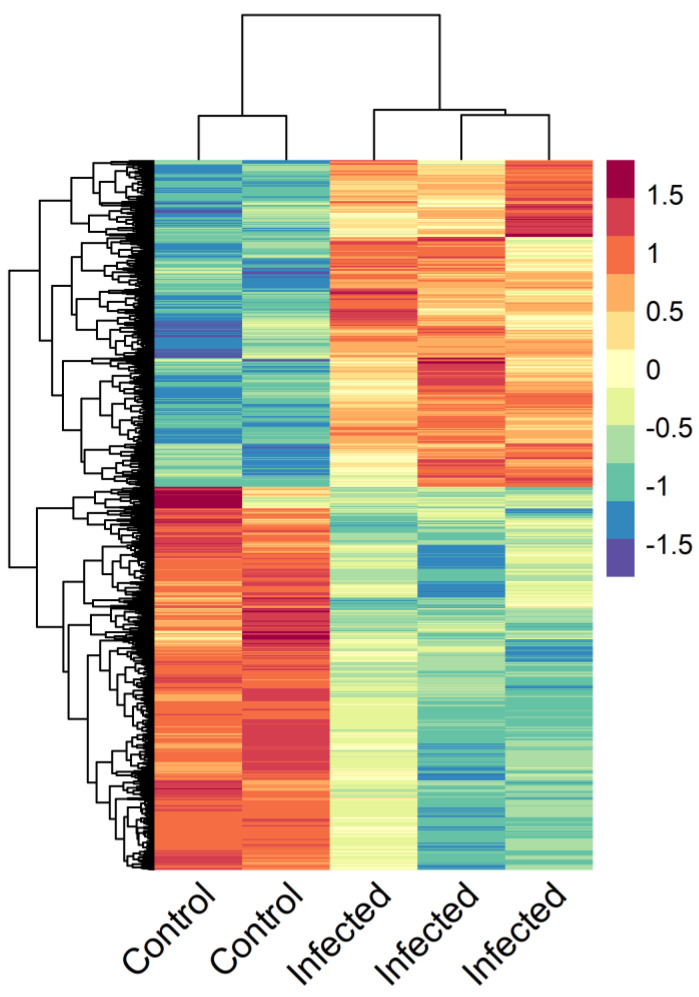
Heat map of the 4640 differentially expressed genes found in thymus tissues of turbot severely infected by *Enteromyxum scophthalmi* compared to controls. Expression values for each gene were scaled from −1.5 to 1.5 by subtracting the mean and dividing by the standard deviation. Samples from infected fish were analyzed individually, while the thymus tissues from four control fish were pooled into two samples for the transcriptomic analysis.

Only four genes showed differential expression between slightly infected and control fish, whereas a total of 4640 differentially expressed genes (DEGs) were detected between the thymus transcriptomes of control and severely infected turbot: 2150 upregulated and 2490 downregulated (Figure 4, Supplementary File 3). 

**Figure 5:**
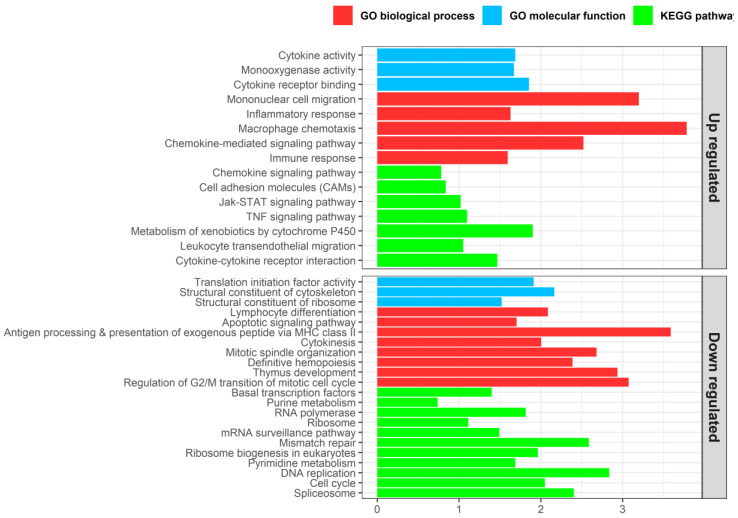
Bar plots of representative enriched gene ontology (GO) biological processes (red), GO molecular functions (blue), and KEGG pathways (green) in the thymus tissues of turbot at advanced stages of infection by *Enteromyxum scophthalmi*. The enrichment analyses were conducted on the sets of up- and downregulated genes found by comparison with healthy specimens. GO terms or pathways showing a Benjamini-Hochberg FDR-corrected *p*-value < 0.05 were considered to be enriched.

GO term and KEGG pathway enrichment analyses were performed to study these DEGs between control and severely infected turbot (Figure 5, Supplementary File 4). Both sets of up- and downregulated genes showed enriched GO terms and KEGG pathways related to immune response. These were mainly associated with inflammatory reaction in the case of upregulated genes (i.e., macrophage chemotaxis, leukocyte transendothelial migration, cytokine activity, TNF signaling pathway), whereas some functions related to immunological role of the organ, such as antigen processing and presentation or lymphocyte differentiation, were found in the enrichment analysis of downregulated genes. The functional analysis also revealed overrepresentation of genes related to protein synthesis and cell proliferation among downregulated genes. Several DEGs related to apoptosis were also found, both up- and downregulated; a GO term related to apoptosis was found to be enriched in the set of downregulated genes (“Apoptotic signaling pathway”), while the “Apoptosis” KEGG pathway was marginally enriched in the upregulated gene set (Benjamini-Hochberg FDR-corrected *p*-value: 0.087, *p*-value: 0.0055; Supplementary File 4). 

## 4. Discussion

The structural and functional characterization of the thymus has scarcely been addressed in many teleost species to date. In particular, the response of the thymus during diseases has barely been studied in cultured species, although several diseases of relevance in aquaculture are known to compromise the immune response and/or cause malnutrition. Infectious diseases and malnourishment are conditions markedly connected to thymus alterations in mammals [9,10,11]. In this study, transcriptomics and histological approaches were employed to contribute to the knowledge of this primary lymphoid organ in turbot, a commercially important species. The histological study ensured the healthy status of control specimens and served to classify *E.-scophthalmi*-infected turbot according to a histopathological grading system [19]. This approach allowed the selection of fish with similar lesions along the time course of experiment for the transcriptomic and immunohistochemical analyses, ensuring the evaluation of the same stage of disease and reducing the intragroup variance and consequently the number of animals needed to attain significant results.

Most of the functions enriched in the thymus tissues of control turbot were related to energy production, protein synthesis, and cell proliferation. This reflects the high anabolic environment of this organ in physiological conditions, also described for terrestrial vertebrates and associated with lymphoblastic proliferation [35]. Accordingly, the immunohistochemical assay using the cell proliferation marker PCNA showed a broad immunoreactivity in the thymus tissues of control fish. As expected, we also found overrepresented functions related to the molecular mechanisms involved in T-cell development (reviewed in Reference [4]): chemokine signaling defines the microenvironments for thymocyte development; antigen processing and presentation and apoptosis act in positive and negative selection of these cells; and T-cell receptor signaling pathways or natural-killer-mediated cytotoxicity can be linked to thymocytes’ definitive maturation as different types of T lymphocytes.

This study was focused on turbot juveniles, which we expected to have fully functional thymuses. In future studies, it would be interesting to assess the potential histological and transcriptomic changes related to aging in turbot. Age-associated thymic involution is a process that apparently occurs in all terrestrial vertebrates [36,37], but it is still unclear to what extent it occurs in fish. The degree of involution seems to be variable among teleosts; some fish species undergo thymic involution in a similar way to mammals, whereas some other species do not show any sign of age-dependent thymic atrophy [6,38]. 

The lesions associated with disease-related effects on thymus tissue and the underlying pathogenic mechanisms are beginning to be understood from studies in human and murine models. Malnutrition and infection by a variety of pathogens, including parasites, have been shown to induce similar atrophy-related alterations in the thymus, consisting of cell depletion, reduction of the cortex:medulla ratio and loss of corticomedullary limit distinction [10,11,39]. This evidence is mainly due to an increased apoptotic rate of immature thymocytes and/or their abnormal egress caused by an impaired selection and export as a consequence of changes in the thymic microenvironment [9,11]. These changes primarily affect chemokines and extracellular matrix (ECM) proteins [11,12,13,14], which are essential components of this microenvironment, with a major role driving thymocyte migration [2,3].

The thymus tissues of turbot infected by *E. scophthalmi* showed intense transcriptomic changes at advanced stages of infection, whereas hardly any changes were noticed in fish with incipient infection, whether with morphological or with transcriptomic techniques. This might be related to a delayed response of the organ to the infection, which is characterized by a long pre-patent period and circumscribed to the gastrointestinal tract [18]. Further, potential parasite-induced silencing of the immune response was postulated at the first stages of infection [21]. 

Turbot enteromyxosis is a serious disease against which the host is incapable of triggering an effective immune response. Previous studies (reviewed in Reference [18]) have shown that an exacerbated and dysfunctional inflammatory response occurs once the parasitic load in the gut increases and the intestinal lesions start to become important. Specifically, high levels of proinflammatory mediators such as TNF-α and iNOS were found in the digestive tract as well as in the immune-related kidney and spleen organs, whereas a poor activation of protective anti-inflammatory mechanisms was observed [20,25,40]. Here, an increased presence of these proinflammatory molecules was observed by immunohistochemistry in the thymus tissues of severely infected fish, and, simultaneously, the transcriptomic profile showed evident signs of activation of the inflammatory response. 

In mammals, pro-inflammatory mediators have been suggested to be responsible for the deleterious effects of infection on the architecture and function of the thymus, inducing apoptosis or alterations of the thymic microenvironment [9,12]. Nitric oxide locally produced through the iNOS signaling pathway has been implicated in thymic atrophy during infection by *Mycobacterium avium* [41], and TNF-α has also been associated with thymic atrophy in Chagas disease, caused by the parasite *Trypanosoma cruzi* [42]. It has also been suggested that TNF-α is involved in the enhanced activity of matrix metalloproteinases (MMPs) in the thymus during infection by *Plasmodium berghei* in mice, which have been related to thymus dysfunction via the alteration of extracellular matrix (ECM) proteins [43]. In that study, an enhanced expression of MMP-2 and MMP-9 and the tissue inhibitors TIMP-1 and TIMP-2 was observed. Here, all the DEGs related to MMPs were upregulated in the thymus tissues of heavily infected turbot, including MMP-2 and TIMP-2 (Supplementary File 3). In general, these results suggest that the thymus is exposed to the deleterious effects of the exacerbated immune response of turbot during enteromyxosis. 

On the other hand, enteromyxosis induces a cachectic syndrome, which is particularly evident in advanced stages of infection, during which fish show severe weight loss and anorexia [18]. Lymphoid atrophy is a well-recognized consequence of nutritional deprivation in humans and animals [10,11,44]. In particular, given the pronounced loss of lymphoid tissue induced by starvation in this organ, the thymus was designated a “barometer of malnutrition” by Prentice [45]. 

The adipocyte-secreted hormone leptin has been indicated as a possible mediator of malnutrition-induced thymic atrophy in mammals [11,12]. Decreased leptin serum levels during malnutrition would compromise its immunoregulatory and anti-apoptotic functions in the thymus, where it acts through the leptin receptors present on thymocytes and reticulo-epithelial cells [39,46,47]. These aspects need to be further studied in teleosts, but most leptin functions appear to be evolutionarily conserved [47]. Interestingly, a gene encoding a leptin receptor was upregulated in the thymus tissues of turbot infected by *E. scophthalmi* (Supplementary File 3). 

Both infectious diseases and malnutrition have been shown to ultimately provoke alterations of chemokines and ECM proteins of the thymic microenvironment [9,12,13,14]. In this study, several genes related to chemokines and ECM proteins governing thymocyte development and migration in mammals [2,3] showed modulated expression in heavily infected turbot (Supplementary File 3). Among these, changes in the expression of C-X-C chemokine receptor type 4, C-C chemokine receptors type 5 and 9, C-C chemokine ligand 25, and C-X-C chemokine ligand 9, and of the ECM components fibronectin and laminin, have been previously related to alteration of the thymic microenvironment in malnourished subject and during infection by *T. cruzi* and *P. berghei* [9,11,13]. 

Regarding apoptosis, the transcriptomic data revealed a modulation of this pathway at advanced stages of enteromyxosis. Moreover, the immunohistochemical technique for the detection of active caspase-3, an effector protease in the apoptotic process, indicated an increased presence of this protein. Thymocytes physiologically undergo apoptosis during the processes of positive and negative selection [48], and our results point towards an increased apoptosis rate in turbot infected by *E. scophthalmi*. However, the mechanisms by which this process is activated and the extent of its role in the development of thymus atrophy should be carefully investigated. A recent study on the thymus tissues of malnourished mice infected by *Leishmania infantum* showed that the alteration of the thymic microenvironment was the main cause underlying thymic atrophy, rather than an increased thymocyte apoptotic rate [13]. Furthermore, these authors demonstrated that the effect of malnutrition on the thymus deeply affects the immune response to this parasite by disturbing T-lymphocyte migration [13,14]. Indeed, both infection and malnutrition can independently give rise to a decline in thymic function that affects the peripheral T-cell pool and determines a negative impact on the ongoing immune response [9,11,12]. In the case of turbot enteromyxosis, we could imagine a scenario where the effect of the proinflammatory mediators and oxidative stress might be enhanced by starvation, preventing a proper immunological response by the thymus and causing the development of atrophy.

Although further investigations on the underlying mechanisms are necessary, strong evidence of declined thymic function during enteromyxosis was found in our study. Many genes found to be highly expressed in the thymus tissues of control fish, and putatively associated with thymic function, were found to be downregulated in infected fish. These genes were related to the immunological role of the organ, such as antigen presentation or lymphocyte differentiation, as well as to the processes of protein synthesis and cell proliferation. The transcriptomic profile was concordant with immunohistochemical and histopathological findings. The immunohistochemistry to PCNA confirmed a minor cell proliferation activity, which is a common feature observed in the thymus during infection and malnutrition [11,14,39]. Furthermore, reduced numbers of cells expressing the T-cell marker CD3ε were found. Most of the severely infected turbot showed cell depletion and altered thymus architecture upon histological examination. All in all, the results suggest a scenario of loss of cellularity and impaired thymic function, consistent with the findings associated with the development of thymus atrophy also observed in other species as a consequence of infectious diseases or dietary deficiencies [10,11]. 

Interestingly, cell depletion is also typically observed in spleen and kidney tissues at advanced stages of enteromyxosis [19]. This observation has been linked to an exacerbated leukocyte recruitment to the intestine, where these cells suffer high apoptotic rates [24,49], as well as to the deleterious effect of a prolonged exposure to proinflammatory cytokines [40]. RNA-seq analysis of those organs in heavily infected turbot also showed a decline in immunological function, as demonstrated by the downregulation of numerous genes related to adaptive immunity and involved in the coordination between innate and adaptive immune responses [20]. The failure in development of a coordinated immune response against *E. scophthalmi* in turbot has been previously associated with the cell depletion suffered by the lymphohematopoietic organs [20,49,50], and this study highlighted that the thymus might also be affected by this phenomenon, which could contribute to the inefficacy of the turbot response against *E. scophthalmi*. It is plausible that common pathogenic mechanisms of cell depletion occur in all lymphoid organs during enteromyxosis, with a probable synergistic effect of inflammatory mediators and malnutrition. A comprehensive understanding of these mechanisms might help to implement therapeutic measures [12,39].

## 5. Conclusions

The present investigation provides the first description of the turbot thymus transcriptome and gene expression profiling integrated with morphological observations. The results indicate the functional relatedness of this organ with that of mammals in physiological conditions. The significant changes found in advanced enteromyxosis suggest that thymic function is strongly affected, pointing towards the development of thymus atrophy as a consequence of the disease. This situation might contribute to the failure of the turbot immune response in fighting the disease. More studies are needed to increase our knowledge of the physiological and pathological thymus microenvironment and to investigate thymus atrophy during diseases in fish. This future research will benefit from combining genomic and proteomic approaches as well as from the identification of further immunohistochemical markers for the characterization of the thymus microenvironment and T-cell subsets. Overall, the present findings highlight that the role of the thymus in pathological conditions should not be overlooked, as it can contribute to a deeper understanding of host–pathogen interactions in infectious diseases. 

## Figures and Tables

**Table 1:** List of the immunohistochemical markers used in this work. AR = antigen retrieval, HUP = heating under pressure.

Marker	Description	Working Conditions	Reference
CD3ε (Donated by Dr. Erin Bromage)	Chain of T-cell co-receptor	1:500 No AR required	[22]
PCNA (M0879, Dako)	Cofactor of DNA polymerase delta	1:500 dilution AR: HUP in pH 6 buffer	[23]
Active caspase-3 (G7481, Promega)	Effector protease in apoptosis	1:200 dilution AR: HUP in pH 8 buffer	[24]
Inos (RB-1605, Thermo Fisher Scientific)	Nitric oxide synthase	1:5000 dilution No AR required	[25]
TNF-α (ab6671, Abcam)	Proinflammatory cytokine	1:600 dilution AR: HUP in pH 6 buffer	[26]

## Supplementary Materials

The following are available online at https://www.mdpi.com/2079-7737/9/8/221/s1, Supplementary File 1: Sequence alignment between the cytoplasmic tail of the CD3ε proteins of rainbow trout (*Oncorhynchus mykiss*) and turbot (*Scophthalmus maximus*). The peptide used by Boardman et al. (2012) as immunogen to produce an anti-rainbow trout CD3ε monoclonal antibody is highlighted in red, and the statistics of the local BLASTp comparison with its turbot homologue are shown. The reference sequences used were NP_001182103.1 (NCBI Reference Sequence) for rainbow trout and ENSSMAT00000000938.1 (Ensembl Transcript ID) for turbot. Supplementary File 2. Healthy thymus GO and KEGG enrichment. Three overlapping subsets of thymus genes were selected based on their normalised levels of expression (genes with TPM > 1000, 100, or 10), and GO term and KEGG pathway enrichment analyses were performed using the whole turbot transcriptome as background. Supplementary File 3. Differentially expressed genes in the thymus of turbot during enteromyxosis. List of genes showing significant differential expression (FDR *p*-value < 0.05) in the thymus at 24 and 42 days postinfection when compared to controls. Supplementary File 4. Enriched GO terms and KEGG pathways in turbot thymus 42 dpi after infection with *Enteromyxum scophthalmi*. List of GO terms and KEGG pathways showing significant enrichment (FDR *p*-value < 0.05) in lists of up- and downregulated genes observed in the thymus at 42 days post infection with *Enteromyxum scophthalmi* when compared to control.
