# Supplementary figures and images for: The Teleost Thymus in Health and Disease: New Insights from Transcriptomic and Histopathological Analyses of Turbot, Scophthalmus maximus
Source: Biology (Basel). 2020 Aug 13;9(8):221. doi: 10.3390/biology9080221 (PMC7465915; doi:10.3390/biology9080221)

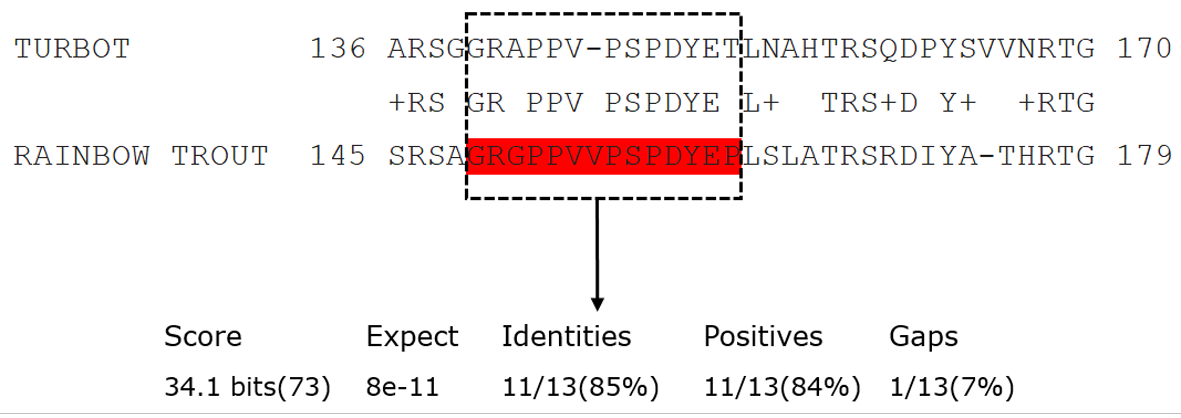

Supplement: Supplementary file 1 [file biology-09-00221-s001.zip › Material suplementario/Supplementary file 1_CD3 sequence alignment.tif]
